# Cell cycle controls stress response and longevity in *C. elegans*

**DOI:** 10.18632/aging.101052

**Published:** 2016-09-25

**Authors:** Matthias Dottermusch, Theresa Lakner, Tobias Peyman, Marinella Klein, Gerd Walz, Elke Neumann-Haefelin

**Affiliations:** ^1^ Department of Medicine IV, Medical Center, Faculty of Medicine, University of Freiburg, Germany

**Keywords:** aging, *C. elegans*, cell cycle, germline, DAF-16/FOXO, SKN-1/Nrf

## Abstract

Recent studies have revealed a variety of genes and mechanisms that influence the rate of aging progression. In this study, we identified cell cycle factors as potent regulators of health and longevity in *C. elegans*. Focusing on the cyclin-dependent kinase 2 (*cdk-2)* and cyclin E (*cye-1*), we show that inhibition of cell cycle genes leads to tolerance towards environmental stress and longevity. The reproductive system is known as a key regulator of longevity in *C. elegans*. We uncovered the gonad as the central organ mediating the effects of cell cycle inhibition on lifespan. In particular, the proliferating germ cells were essential for conferring longevity. Steroid hormone signaling and the FOXO transcription factor DAF-16 were required for longevity associated with cell cycle inhibition. Furthermore, we discovered that SKN-1 (ortholog of mammalian Nrf proteins) activates protective gene expression and induces longevity when cell cycle genes are inactivated. We conclude that both, germline absence and inhibition through impairment of cell cycle machinery results in longevity through similar pathways. In addition, our studies suggest further roles of cell cycle genes beyond cell cycle progression and support the recently described connection of SKN-1/Nrf to signals deriving from the germline.

## INTRODUCTION

The nematode *Caenorhabditis elegans* has been invaluable to biological research of mechanisms that slow aging processes and may prevent age-related diseases. *C. elegans* is well suited for molecular studies of aging because of its easy genetic tractability and lifespan of less than 3 weeks. Aging has been linked to a variety of different signaling pathways, most notably the reproductive system. Removing the germline of *C. elegans* extends lifespan by up to 60% [1]. The role of germline signaling in the regulation of health and longevity has been shown to be evolutionarily conserved in several species, including *C. elegans*, *D. melanogaster,* and mice [1-4]. Also, in humans it has been suggested that the reproductive state and lifespan correlate [5, 6]. The lifespan extension associated with ablation of the germline in *C. elegans* is caused specifically by loss of proliferating germline stem cells and requires the preservation of the somatic gonad [1, 7]. These findings suggest that longevity is not simply a result of sterility but is regulated by counterbalancing signals produced by the germline and somatic gonad. In *C. elegans*, the effects of germline absence on longevity have been studied either by laser ablation of germline precursor cells or by genetic mutants that show defects in germ cell proliferation. The most studied gene in this context is *glp-1*, which encodes a homolog of Notch that is expressed in germline stem cells and whose normal role is to promote proliferation of germ cells [8]. Mutation of *glp-1* reduces the number of germline stem cells and thus promotes longevity [7].

The FOXO-family transcription factor DAF-16 is needed for germline removal to extend lifespan [1]. DAF-16 is best known for its ability to promote longevity in response to reduced insulin/insulin-like growth factor 1 (IGF-1) signaling [reviewed in 9]. However, the mechanisms by which insulin/IGF-1 signaling and germline loss activate DAF-16 seem to be distinct. For example, KRI-1/KRIT ankyrin repeat protein and the TCER-1/TCERG1 transcription elongation factor are required for germline absence to induce DAF-16 nuclear accumulation and promote longevity but are not involved in insulin/IGF-1 signaling [10, 11]. Moreover, loss of germ cells further increases the lifespan of already long-lived insulin pathway mutants [1]. Ablation of the germline leads to DAF-16 activation and accumulation primarily in nuclei of intestinal cells. The intestine seems to play a key role in this pathway, as expression of DAF-16 exclusively in this tissue is sufficient to extend lifespan in germline-less animals [12]. Steroid hormone signaling also plays an important role for gonadal longevity. In germline-deficient animals, the nuclear hormone receptor DAF-12 and DAF-9, a cytochrome P450 synthesizing DAF-12 ligands, stimulate nuclear accumulation of DAF-16 and promote longevity [10, 13].

Genetic experiments revealed that longevity signaling from the reproductive system involves several other transcription factors in addition to DAF-16. Recently, the transcription factor SKN-1, orthologous to mammalian Nrf (NF-E2 related factor) proteins has been implicated in long life from germline-less animals [14-16]. SKN-1 mediates a wide range of oxidative stress defense, detoxification, has important metabolic functions, and promotes longevity in various species [reviewed in 17].

The cell cycle is a well-coordinated set of events culminating in cell growth and division. Evolutionarily conserved regulators of this process include cyclins, cyclin-dependent kinases (CDKs) and CDK inhibitors (CKI). CDKs partner with regulatory subunits, the cyclins, which control kinase activity and substrate specificity. CDK/cyclin complexes thus ensure sequential progression through the cell cycle in an ordered fashion [reviewed in 18]. A key regulator for progression of the cell cycle from the G1 to the S phase is the CDK2/cyclin E complex. Once activated, this complex phosphorylates and therefore inhibits the retinoblastoma protein Rb, hence releasing the E2F transcription factor which activates gene expression for cell cycle progression [19-21].

In addition to their main functions in cell cycle control, recent research has indicated that mammalian CDKs, cyclins, and CKIs play diverse roles in a variety of cellular processes such as transcription, DNA-damage repair, epigenetic regulation, metabolism, proteolytic degradation, and stem cell self-renewal [reviewed in 22]. Interestingly, CDKs and cyclins can accomplish these functions at least in part without complex formation. Notably, studies in *C. elegans* revealed important functions of cell cycle regulators during development beyond their traditional role in cell cycle. CDK-1 and cyclin B contribute to transition from oocyte to embryo, asymmetric cell division, and cell fate specification by regulating the localization and timely elimination of cell fate determinants [23-25]. CDK-2 and cyclin E have been shown to control the balance between mitotic proliferation and meiotic differentiation in the *C. elegans* germline by reducing abundance of the GLD-1 translational repressor [26, 27]. CDC25 phosphatases are key positive cell cycle regulators through their ability to remove inhibitory phosphate from CDK/cyclin complexes [28]. Intriguingly, knockdown of *cdc-25* in adult *C. elegans* has been shown to promote stress tolerance and longevity [29]. However, the molecular mechanisms how *cdc-25* influences aging remain to be elucidated. Together, these studies suggest that CDKs, cyclins, and regulatory proteins can also influence cellular and developmental processes in addition to the cell cycle.

The genetic evidence that CDC-25 is important for stress response and aging raises the question of whether further cell cycle regulators or the entire cell cycle machinery may have broader roles in these processes. Here, we have investigated how longevity is affected by genetic inhibition of *cdk-2* and cyclin E (encoded by *cye-1*) in *C. elegans*. We find that heat tolerance, oxidative stress resistance, and longevity are increased when *cdk-2* and *cye-1* are inhibited. We localize this effect to the germline and identify that germline signaling plays a central role in its influence on aging. SKN-1 and DAF-16 activate protective gene expression and extend lifespan when *cdk-2/cye-1* is inactivated. Genetic interference with further cell cycle genes also increases lifespan and triggers a similar SKN-1-dependent response, suggesting the notion that cell cycle factors might influence aging through their regulatory functions in germline proliferation.

## RESULTS

### Inhibition of cell cycle regulates adult lifespan in *C. elegans*

**Figure 1:**
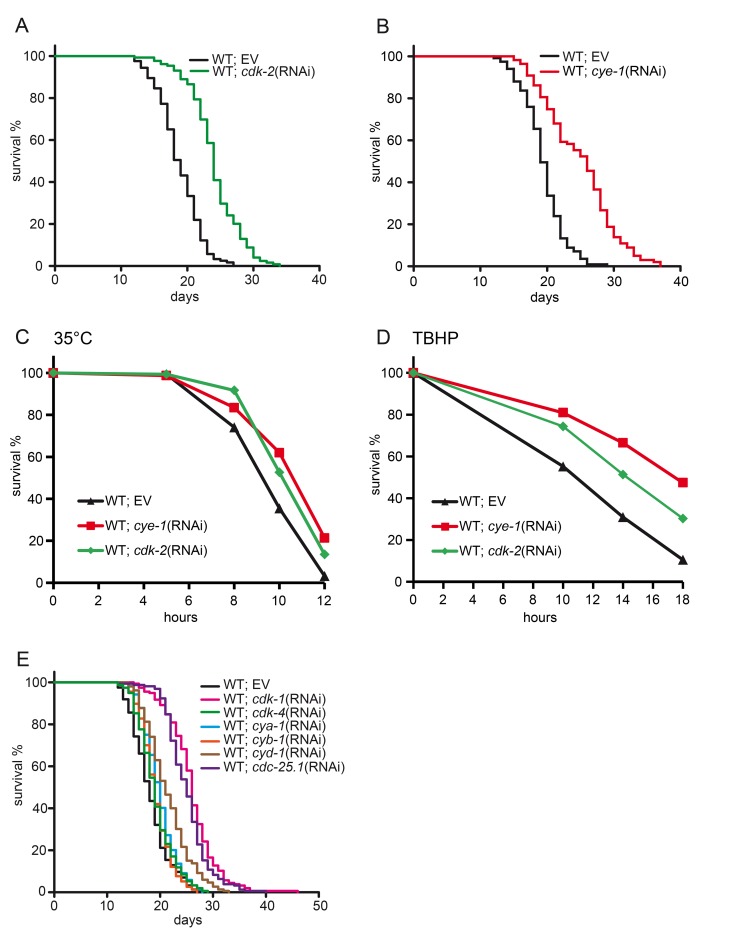
Inhibition of cell cycle extends lifespan and enhances stress tolerance (**A**) Lifespan analysis in wild type animals fed with *cdk-2*(RNAi) or control. Inhibition of *cdk-2* increases the mean lifespan by 28 %. (**B**) Lifespan analysis in wild type animals fed with *cye-1*(RNAi) or control. (**C**) Increased resistance to heat (35°C) deriving from *cdk-2* and *cye-1*(RNAi). See Table S3 for statistics. (**D**) Inhibition of *cye-1* and *cdk-2* by RNAi increases resistance to oxidative stress from TBHP. Statistical analyses are presented in Table S4. (**E**) Inhibition of key components of the cell cycle machinery extends lifespan. EV refers to empty RNAi vector control. All survival plots show combined data from at least two experiments. See also Table 1 for corresponding data and statistics, and Table S1 for individual experiments.

To assess the function of cell cycle factors in regulation of longevity we first knocked down the cyclin-dependent kinase *cdk-2* by RNA interference (RNAi). We used RNAi feeding starting from L4/early adulthood to inhibit *cdk-2* and obtain results that were not confounded by its developmental functions. We found that post-developmental inactivation of *cdk-2* had a strong effect on lifespan with an increase of 28% compared to wild type control (24.1 d in *cdk-2*(RNAi) vs 18.8 d control (RNAi)) (Figure 1A and Table 1). Thus, inhibition of *cdk-2* function during adulthood is sufficient to confer longevity. The activity of cyclin-dependent kinases (CDKs) and their substrate specificity is regulated by cyclins. CDK-2 acts in concert with the cyclin E homolog CYE-1 to govern mitotic cell cycle [reviewed in 30]. Therefore we next tested knockdown of *cye-1*. *cye-1*(RNAi) resulted in lifespan extension similar to *cdk-2*(RNAi) (24.9 d in *cye-1*(RNAi) vs 19.5 d *control*(RNAi)) (Figure 1B and Table 1).

**Table 1:** Lifespan analyses. Pooled lifespan data shown in Figures 1-3

Strain	RNAi	Mean lifespan ± SEM (days)	Median lifespan (days)	75^th^ Percentile	25^th^ Percentile	p value vs. N2 (control)	p value vs. mutant	% mean lifespan change vs. N2 (control)	% mean Lifespan change vs. mutant	N	No.of Exp	Figure
N2	control	18,8±0,3	19	21	17					123/129	2	1A
N2	*cdk-2*	24,1±0,3	24	26	22	2,0E-27		28%		125/141	2	1A
N2	control	19,5±0,3	19	21	18					114/120	2	1B
N2	*cye-1*	24,9±0,5	26	29	20	5,7E-19		28%		103/120	2	1B
N2	control	18,2±0,3	18	20	15					158/161	2	1E
N2	*cdk-1*	26±0,4	26	28	23	9,6E-46		43%		157/160	2	1E
N2	*cdk-4*	19,2±0,3	19	21	17	0,035		6%		153/160	2	1E
N2	*cya-1*	19,9±0,3	20	22	18	2,9E-04		10%		155/160	2	1E
N2	*cyb-1*	19,2±0,2	19	21	17	0,087		6%		157/160	2	1E
N2	*cyd-1*	21,5±0,3	21	24	18	6,7E-12		18%		153/160	2	1E
N2	*cdc-25.1*	25,1±0,3	25	27	22	1,2E-41		38%		158/160	2	1E
N2	control	19±0,2	19	21	17					267/275	4	2A
N2	*cye-1*	26,1±0,3	26	29	24	6,8E-84		38%		247/270	4	2A
*glp-1(e2141)*	control	23,9±0,3	24	27	21	7,0E-54		26%		315/413	4	2A
*glp-1(e2141)*	*cye-1*	24,5±0,3	24	28	21	1,8E-58	0,075 vs *glp-1*(ctr)	29%	3%	305/401	4	2A
N2	control	18,9±0,3	19	21	16					122/124	2	2B
N2	*cye-1*	24,2±0,4	24	26	22	8,8E-20		28%		105/113	2	2B
*mes-1(bn7)* (fertile)	control	18,2±0,4	18	20	15	0,31		−4%		116/122	2	2B
*mes-1(bn7)* (fertile)	*cye-1*	22,1±0,5	22	25	19	6,9E-08	1,9E-09 vs *mes-1*F(ctr)	17%	21%	84/97	2	2B
*mes-1(bn7)* (sterile)	control	24,2±0,7	23	28	19	1,4E-12		28%		88/115	2	2B
*mes-1(bn7)* (sterile)	*cye-1*	23,7±0,7	23	27	18	1,5E-10	0,84 vs *mes-1*S(ctr)	25%	−2%	90/111	2	2B
N2	control	19,9±0,3	20	23	17					110/111	2	2C+2D
N2	*cye-1*	24±0,3	24	26	22	3,8E-14		21%		102/118	2	2C+2D
*daf-12* *(rh61rh411)*	control	17,6±0,3	18	20	15	4,5E-07		−11%		147/147	2	2C
*daf-12* *(rh61rh411)*	*cye-1*	19,3±0,3	19	22	17	0,38	1,9E-05 vs *daf-12*(ctr); 9,9E-017 vs N2(*cye-1)*	−3%	10%	133/149	2	2C
*daf-9(rh50)*	control	18,5±0,4	18	22	16	0,026		−7%		128/130	2	2D
*daf-9(rh50)*	*cye-1*	19,7±0,4	20	23	16	0,51	9,7E-03 vs *daf-9(*ctr); 9,8E-021 vs N2(*cye-1)*	−1%	6%	123/123	2	2D
N2	control	19,4±0,3	19	21	16					129/134	2	3A+3D
N2	*cye-1*	25,3±0,4	25	28	23	9,2E-28		30%		127/136	2	3A+3D
*daf-16 (mgDf47)*	control	17,2±0,3	17	20	14	4,3E-05		−11%		161/169	2	3A
*daf-16 (mgDf47)*	*cye-1*	19,2±0,4	19	23	15	0,29	8,3E-06 vs *daf-16*(ctr)	−1%	11%	168/188	2	3A
*skn-1(zu67)*	control	16,1±0,3	16	18	14	2,8E-10		−17%		132/135	2	3D
*skn-1(zu67)*	*cye-1*	15,7±0,3	16	18	13	7,0E-12	0,53 vs *skn-1*(ctr)	−19%	−3%	124/129	2	3D
N2	control	19,4±0,3	19	21	17					115/123	2	3B
N2	*cye-1*	25,5±0,3	25	28	23	6,3E-29		31%		113/139	2	3B
*kri-1(ok1251)*	control	19±0,2	19	21	17	0,42		−2%		149/154	2	3B
*kri-1(ok1251)*	*cye-1*	21±0,4	20	24	18	7,7E-04	7,1E-06 vs *kri-1*(ctr)	8%	10%	113/131	2	3B
N2	control	19,3±0,3	19	22	17					106/139	2	3C
N2	*cye-1*	25±0,4	24	28	22	4,4E-19		29%		75/130	2	3C
*daf-2(e1370)*	control	52,3±0,8	55	60	47	4,1E-83		171%		179/190	2	3C
*daf-2(e1370)*	*cye-1*	58,2±0,8	59	66	54	7,4E-90	9,1E-010 vs *daf-2*(ctr)	202%	11%	178/199	2	3C

These combined results were derived from individual experiments that are described in Supplementary Table S1. Experiments are grouped and graphed in the indicated figures. Wild type N2 animals were used for RNAi treatment. SEM: standard error of the mean. 75^th^ and 25^th^ percentiles refer to the day at which 75% or 25% of the analyzed population is dead. N represents number of observed deaths / total number of worms in experiment. P values were calculated by log-rank test.

To determine whether the timing of cell cycle gene knockdown might influence lifespan, we initiated *cye-1* RNAi feeding either during development (starting from L1 throughout life), in L4, or in adulthood (day 3) when the worms produced eggs. Interestingly, we found that knockdown of *cye-1* during development as well as in adulthood resulted in longevity (Supplementary Figure 1A and Table S2) suggesting that cell cycle events in the larvae may also modulate the aging process. Furthermore, inhibition of progeny production by supplementation of fluorodeoxyuridine (FUDR) did not affect the longevity of *cye-1*(RNAi). We obtained comparable results with or without inclusion of FUDR (Supplementary Figure 1B and Table S2).

Many *C. elegans* mutations and manipulations that increase lifespan also confer resistance towards diverse forms of stress [31]. We analyzed heat and oxidative stress response of *cye-1* and *cdk-2* deficient worms. Knockdown of *cye-1* and *cdk-2* by RNAi enhanced tolerance to thermal stress (Figure 1C) and oxidative stress (Figure 1D) compared to wild type control.

*C. elegans* displays age-related degenerative changes including reduction of pharyngeal pumping and body movement during its lifespan [32]. There is a positive correlation between the decline of neuromuscular behavior and survival probability. We therefore assessed whether inhibition of cell cycle genes could also attenuate the decline of locomotory functions. We observed that *cye-1*(RNAi) and *cdk-2*(RNAi) delayed the age-related decline of pharyngeal pumping (Supplementary Figure 1C) and body movements (Supplementary Figure 1D). We also quantified the number of progeny after *cye-1* and *cdk-2* knockdown by RNAi. Consistent with essential roles of *cye-1* and *cdk-2* for cell cycle progression and embryonic develop-ment, *cye-1*(RNAi) and *cdk-2*(RNAi) significantly reduced the number of progeny compared to wild type (Supplementary Figure 1E), although the effect was only partially penetrant. We further assessed the morphology of the gonad after 2 generations of RNAi feeding against *cye-1* and *cdk-2*, and observed that a portion of the animals were sterile or even arrested during larval development (Supplementary Figure 1F and 1G).

Given the profound impact that inhibition of *cye-1* and *cdk-2* had on longevity, we wondered if other components of the cell cycle also control aging. We therefore tested activators of the cell cycle including the cyclin-dependent kinases *cdk-1* and *cdk-4,* their cyclin partners *cya-1, cyb-1* and *cyd-1,* and the CDK-activating phosphatase *cdc-25.1*. We observed that RNAi-knockdown of these core components of the cell cycle machinery during adulthood significantly extended lifespan (Figure 1E and Table 1). We focused our analyses on *cdk-2* and the corresponding cyclin *cye-1* because they are well described key regulators of the cell cycle and robustly extended lifespan.

### Cell cycle genes regulate longevity through the germline

**Figure 2:**
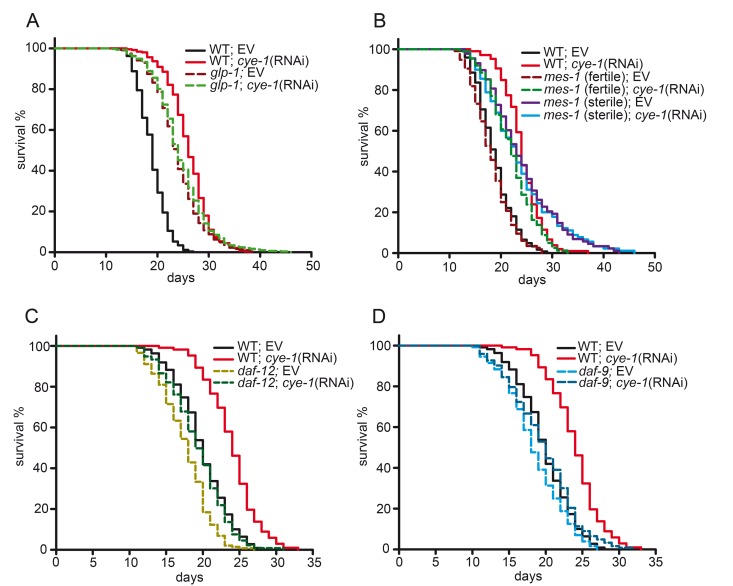
Cell cycle genes function in the germline longevity pathway (**A**) *cye-1*(RNAi) does not alter longevity of germline-defective *glp-1(e2141)* mutants. *glp-1* mutants were raised at 25°C until L4/early adulthood to eliminate germ cells and then shifted to 20°C for the rest of the assay. (**B**) Lifespans of germline-defective *mes-1* mutants. *mes-1(bn7) induced sterility is approximately 50% penetrant at 20°C [7].* The sterile and fertile *mes-1* animals were identified by their appearance using a dissecting microscope. *cye-1*(RNAi) extends lifespan in fertile *mes-1* animals but not in sterile *mes-1* mutants. (**C**) The steroid hormone signaling pathway is required for *cye-1*(RNAi) longevity. The nuclear hormone receptor *daf-12(rh61rh411)* mutants greatly suppress the longevity of *cye-1*(RNAi) treated worms. (**D**) Inactivation of the steroid hormone biosynthesis enzyme *daf-9* abolishes lifespan extension caused by *cye-1*(RNAi). Survival plots show combined data from at least two experiments. Quantitative data and statistical analyses of lifespan assays are shown in Table 1, individual experiments are presented in Table S1. EV refers to empty control vector.

To determine if an intact germline is necessary for lifespan regulation by cell cycle genes, we examined the effect of *cye-1* and *cdk-2* knockdown in *glp-1* mutant worms. *glp-1* encodes a Notch family receptor that is essential for mitotic proliferation of germline stem cells [33, 34]. We used the *glp-1* temperature-sensitive mutant allele *e2141*. When grown at the non-permissive temperature of 25°C, these mutants lack the germline and are long lived [7, 34]. We observed that knockdown of *cye-1* and *cdk-2* by RNAi did not further extend the longevity of *glp-1(e2141)* mutant worms (Figures 2A and S2A, Table 1 and S2) suggesting that cell cycle genes require the germline to regulate longevity. We found that this effect could be reproduced by another germline-defective mutant, *mes-1(bn7).* The *mes-1* gene controls initial development of the embryonic germline [35]. At 20°C, about 50% of *mes-1(bn7)* mutants lack the germline, and these sterile animals live up to 50% longer than their fertile siblings [7]. Knockdown of c*ye-1*(RNAi) was able to extend the lifespan of fertile *mes-1* worms (21%), but not sterile *mes-1* animals (Figure 2B and Table 1). We further assessed the function of cell cycle regulators specifically in the germline using *rrf-1* mutants. RRF-1 is mainly required for RNAi in somatic tissues. Hence, in animals lacking *rrf-1* activity RNAi is efficient in the germline but it functions poorly in somatic cells [36]. We found the *cye-1(RNAi)* strongly extended lifespan in *rrf-1(pk1417)* mutants (18% compared to *rrf-1* mutants fed with control(RNAi)). The effect was comparable to wild type (Supplementary Figure 2B and Table S2). Together, these findings indicate that cell cycle genes function in the germline to influence lifespan.

The longevity of animals lacking the germline depends on steroid hormone signaling pathways. Mutations in the nuclear hormone receptor *daf-12* prevent the lifespan-extending effect when germline stem cells are removed [1, 10, 37]. To test whether *cye-1* genetically interacts with *daf-12* to regulate lifespan, we examined the effect of *cye-1* knockdown in the *daf-12(rh61rh411)* null mutant. We found that *daf-12(rh61rh411)*;*cye-1*(RNAi) animals have significantly shortened lifespans compared with *cye-1*(RNAi) (Figure 2C and Table 1). Enzymes that produce DAF-12 ligands, such as the cytochrome P450 DAF-9 also contribute to steroid hormone signaling and mutation of *daf-9* abrogates the longevity of germline-less animals [13, 37]. The loss of function mutant *daf-9(rh50)* displays a slightly reduced lifespan, presumably because DAF-12 is not fully activated [37]. We observed that the lifespan extension of *cye-1*(RNAi) was strongly suppressed in the *daf-9* mutant background (Figure 2D and Table 1). Together, these observations suggest that *cye-1* exerts its effect on lifespan through the steroid hormone signaling pathway including DAF-9 and the nuclear hormone receptor DAF-12.

### Longevity associated with inhibition of cell cycle requires DAF-16

**Figure 3:**
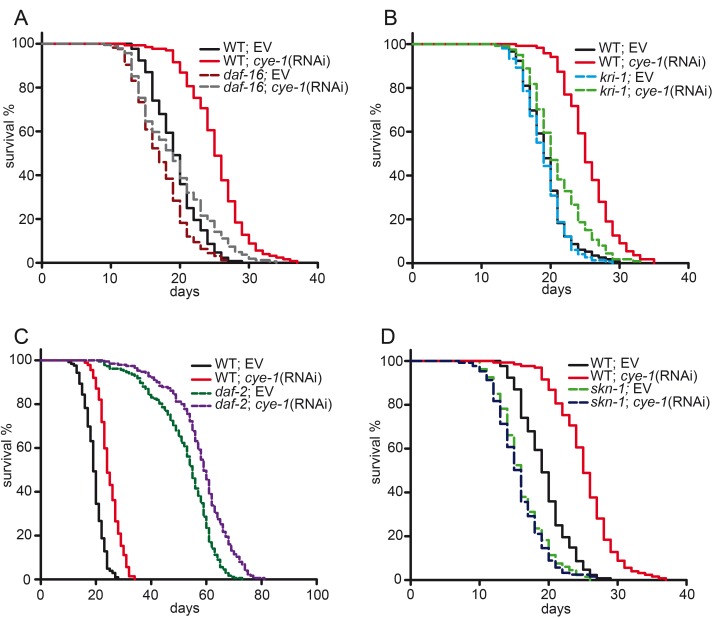
DAF-16 and SKN-1 are required for longevity from reduced cell cycle activity (**A**) Lifespan extension in response to cell cycle inhibition is mediated by DAF-16. *daf-16(mgDf47)* null mutants and wild type worms were fed with *cye-1* and *control*(RNAi). The longevity associated with *cye-1*(RNAi) was greatly decreased but not eliminated by *daf-16* mutation. (**B**) Longevity of *cye-1*(RNAi) was greatly reduced by *kri-1(ok1251)* mutants. (**C**) Knockdown of *cye-1* by RNAi further increases longevity of *daf-2(e1370)* mutants. (**D**) Longevity extension by *cye-1(RNAi)* was eliminated by *skn-1(zu67)* mutation. Survival plots show combined data from at least two experiments and are summarized in Table 1, individual experiments are presented in Table S1. EV refers to empty control vector.

It is well established that the activity of the transcription factor DAF-16/FOXO is required for germline removal to extend lifespan, as *daf-16* mutants completely abolish longevity of germline-less animals [1]. Upon germline elimination DAF-16 accumulates in the nucleus of intestinal cells and stimulates expression of target genes [12, 38, 39]. We first analyzed the role of cell cycle genes in the DAF-16-dependent germline longevity pathway. We found that the *daf-16(mgDf47)* null mutation [40] significantly suppressed the longevity of *cye-1*(RNAi) (Figure 3A and Table 1) and *cdk-2*(RNAi) treated worms (Supplementary Figure S2C and Table S2). Interestingly, the *daf-16(mgDf47)* mutation did not fully block the pro-longevity effect of cell cycle inhibition. RNAi against *cye-1* and *cdk-2* produced a modest but consistent increase in the mean lifespan of the *daf-16* mutant (11% and 10%, respectively), but to a much lesser extent than in wild type (30% and 28%) (Table 1 and S2). We tested the genetic interaction with a different *daf-16* null allele: again knockdown of *cye-1* slightly extended the mean lifespan of *daf-16(mu86)* mutants (14% compared to 23% in wild type) (Supplementary Figure S2D and Table S2). These findings indicate that the longevity benefits of cell cycle inhibition largely depend upon DAF-16 but also raise the possibility that a different mechanism might be involved.

The ankyrin-repeat protein KRI-1/KRIT1 is needed for loss of the germline to target DAF-16 to the nucleus and extend lifespan [10]. Next, we investigated if *kri-1* is also required for the longevity of cell cycle deficient worms. We observed that the longevity associated with *cye-1*(RNAi) and *cdk-2*(RNAi) was strongly suppressed by lack of *kri-1,* shortening mean lifespan by up to 18% (Figure 3B and S2E, Tables 1 and S2).

The reproductive pathway functions in a synergistic manner with the *daf-2*/insulin-like signaling pathway to regulate DAF-16, as loss of the germline greatly extends the long lifespan of *daf-2* mutants [1]. Accordingly, we observed that knockdown of *cye-1*(RNAi) in *daf-2(e1370)* mutants further increased their longevity (Figure 3C and Table 1) indicating that cell cycle genes act in the DAF-16-dependent germline longevity pathway but not in the insulin signaling pathway.

### SKN-1/Nrf is required for longevity from reduced cell cycle activity

It was intriguing that *daf-16* null mutants did not completely suppress longevity associated with inactivation of *cye-1* and *cdk-2.* Our findings suggested that inhibition of the cell cycle might affect longevity through a pathway in parallel to DAF-16. The transcription factor SKN-1/Nrf has conserved functions in stress defense, protein homeostasis, and metabolism and promotes longevity [41, 42]. Recently, SKN-1 has been shown to promote longevity in the absence of germline stem cells [15, 16]. We wondered whether SKN-1 might act in parallel to DAF-16 for longevity under conditions in which cell cycle processes are inactive. We therefore tested the effects of *cye-1* and *cdk-2* RNAi knockdown in the *skn-1(zu67)* mutant and wild type, and found that the pro-longevity effect of *cye-1* and *cdk-2*(RNAi) was essentially prevented by mutation of *skn-1* (Figure 3D and Supplementary Figure S2F, Table 1 and S2). These observations indicate that SKN-1 is fully required for the effects of cell cycle factors on longevity.

### Cell cycle inhibition induced a transcriptional stress response that involves SKN-1 and DAF-16

**Figure 4:**
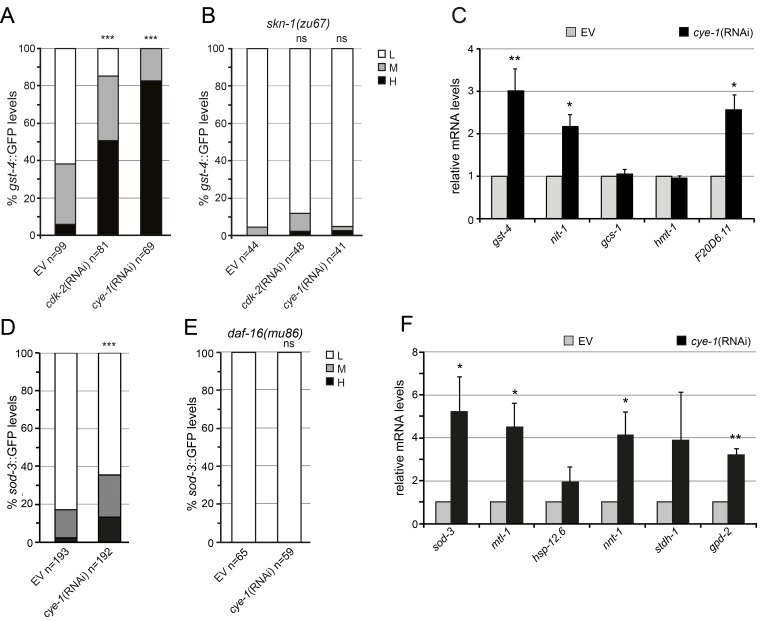
Cell cycle inhibits SKN-1 and DAF-16-driven stress response (**A**) Activation of the *Pgst-4*::GFP reporter in L4/young adult animals that were exposed to *cye-1*(RNAi), *cdk-2*(RNAi), or control beginning from L1. (**B**) Analysis of *Pgst-4:*:GFP expression in *skn-1(zu67)* mutants. In (A) and (B) induction of the *Pgst-4*::GFP reporter in the intestine was quantified as low (L), medium (M) and high (H) (see Supplementary Figure S3C). Pooled data from 2 experiments. *p*-values were calculated by the Chi-square test. ***p < 0.001. ns, not significant. n, number of worms analyzed. (**C**) Induction of endogenous SKN-1 target gene expression in response to *cye-1*(RNAi) analyzed by qPCR. RNAi was performed from L4 to day four of adulthood. Data are presented as fold change compared to wild-type on *control*(RNAi) averaged from at least three independent experiments, error bars represent SEM. *p*-values were derived from a student's t-test. *p< 0.05. **p<0.01. (**D**) *cye-1*(RNAi) induces expression of *Psod-3*::GFP (see also Supplementary Figure S4). (**E**) Analysis of *Psod-3*::GFP expression in *daf-16(mu86)* mutants. In (D) and (**E**) *Psod-3*::GFP quantification with high (H), medium (M), and low (L) scoring. Pooled data from at least 2 experiments. *p*-values were calculated by the Chi-square-test. ***p < 0.001. ns, not significant. n, number of worms analyzed. (**F**) DAF-16 target gene expression assayed by qPCR. Worms were exposed *to cye-1*(RNAi) or empty vector control during adulthood. The *nnt-1*, *stdh-1* and *gpd-2* genes have been shown previously to be upregulated by germline removal. Data are mean ± SEM. *p*-values were derived from a student's t-test. *p< 0.05. **p<0.01.

Our results raise the question if the activity of SKN-1 is actually controlled by inhibition of the cell cycle. SKN-1 accumulates in intestinal nuclei and upregulates expression of detoxification genes in response to certain stresses or when mechanisms that limit SKN-1 activation are inhibited [41-44]. We further investigated how RNAi knockdown of cell cycle factors activates SKN-1 dependent transcriptional response and analyzed the expression of the SKN-1 target gene glutathione S-transferase, *gst-4* using a transcriptional GFP reporter (*Pgst-4:*:GFP) [45]. In the intestine the *Pgst-4:*:GFP reporter was expressed at very low levels under normal conditions (Figure 4A). Transcription from the transgenic *gst-4* promoter was robustly induced by RNAi knockdown of *cye-1* and *cdk-2* (Figure 4A). This *gst-4* induction was greatly abolished by mutation of *skn-1* (Figure 4B). Similarly, inhibition of the cyclin *cya-1, cyb-1,* and *cyd-1*, the cyclin-dependent kinases *cdk-1* and *cdk-4*, and the CDK activator *cdc-25.1* resulted in robust *gst-4* activation through *skn-1*-dependent mechanisms (Supplementary Figure 3A-C).

We next analyzed effects on endogenous SKN-1–regulated gene expression in adult worms after inhibition of *cye-1* through RNAi. Here, we focused on *cye-1* representative for different cell cycle factors. We used quantitative PCR to analyze the mRNA levels of a set of well characterized SKN-1 targets involved in various stress processes like glutathione S-transferase *gst-4*, γ-glutamyl cysteine synthetase *gcs-1*, oxidoreductase F20D6.11, nitrilase *nit-1*, and ABC transporter *hmt-1* [42, 43]. mRNA production of *gst-4* and *nit-1* has also been shown to depend on SKN-1 under normal (unstressed) conditions, while *gcs-1*, *hmt-1*, and F20D6.11 are upregulated by SKN-1 in response to stress [42]. We observed that RNAi knockdown of *cye-1* increased the expression of several endogenous SKN-1 target genes in adult worms (Figure 4C). We conclude that inhibition of cell cycle factors promotes the transcriptional activity of SKN-1 for detoxification and stress defense.

Given the importance of DAF-16 for longevity associated with cell cycle inhibition we investigated whether *cye-1* influences the expression of DAF-16 target genes. First, we analyzed the expression of the conserved DAF-16/FOXO target superoxide dismutase (*sod-3*). In adult worms, a *sod-3*::GFP transgenic reporter was expressed at low levels under normal conditions, but was elevated by *cye-1*(RNAi) (Figure 4D). The up-regulation of *sod-3* was strongly attenuated in *daf-16* mutants (Figure 4E). Next, we used qPCR to analyze how knockdown of *cye-1* affected the transcriptional profile of well-identified DAF-16 target genes [46, 47]. Several stress resistance genes like *sod-3*, *mtl-1* encoding a metallothionein protein, and *hsp-12.6* encoding a small heat shock protein were upregulated under *cye-1*(RNAi) conditions (Figure 4F). We also analyzed the expression of genes known to be upregulated by germline removal, including *gpd-2*, *stdh-1* and *nnt-1* [11]. We found that the expression of these genes was strongly induced by *cye-1*(RNAi) knockdown (Figure 4F). Together, our data indicate that when cell cycle factors are inhibited, SKN-1 and DAF-16 induce a protective transcriptional response.

## DISCUSSION

The last few decades have unraveled a variety of different mechanisms and signaling pathways that actively influence aging in *C. elegans*. Focusing on cyclin E *cye-1* and cyclin-dependent kinase 2 *cdk-2*, we here characterize a yet unknown role of cell cycle genes in the regulation of longevity, health and stress resistance (Figure 1). Additionally, inactivation of several other core components of the cell cycle machinery similarly extended lifespan (Figure 1E) supporting the idea that cell cycle regulators play a role for longevity in *C. elegans*.

Several lines of evidence suggest that these longevity benefits are mediated through specific regulatory pathways involving the germline. Proliferating cells are the likeliest target in this mechanism and the germline is the only tissue in adult *C. elegans* with a continuously proliferating pool of cells, whereas somatic tissues are entirely post-mitotic [48]. Consistent with this idea, genetic inhibition of *cdk-2* and *cye-1* did not further extend the lifespan of *glp-1* mutants in which the number of germline stem cell is markedly reduced (Figure 2A and Supplementary Figure 2A). Similarly, inactivation of *cye-1* did not increase longevity in *mes-1* mutants that fail to develop a germline and are sterile, by contrast to results obtained in fertile *mes-1* mutants (Figure 2B). Moreover, *cye-1*(RNAi) increased lifespan in *rrf-1* mutants in which RNAi-mediated gene knockdown functions most efficiently in the germline (Supplementary Figure 2B). Importantly, the longevity effect was still apparent when RNAi-mediated *cye-1* knockdown was initiated at different stages, during larval development or later adulthood (day 3) (Supplementary Figure 1A). Of note, lifespan can be extended in *C. elegans* when germline proliferation is inhibited by *glp-1* inactivation during adulthood [7]. In flies, similar findings have been described [3]. Together, our data show that the benefits associated with genetic inhibition of cell cycle regulators involve germline mechanisms.

It is well established that germline-mediated longevity requires the activity of the FOXO transcription factor DAF-16 and a lipophilic hormone/steroid signaling pathway comprising DAF-9 and DAF-12 [1, 7, 13, 37]. We found that these canonical germline pathway components also play a critical role in the increases in lifespan that derive from cell cycle inhibition (Figure 2C and D, Figure 3A, Supplementary Figure 2C and D). Interestingly, the dramatic lifespan extension associated with knockdown of *cye-1* and *cdk-2* was not fully eliminated in two *daf-16* null mutants indicating that DAF-16 is central for the influence of cell cycle regulators on longevity, but additional factors contribute to those effects. Inhibition of *cye-1* by RNAi led to induction of DAF-16 target genes (*gpd-2*, *stdh-1* and *nnt-1*) (Figure 4D-F) whose expression is known to be changed following germline-loss [11]. Previous work has shown that KRI-1 is needed for loss of germline to trigger DAF-16 activity and extend lifespan [10]. We observed that the increases in lifespan that derive from cell cycle inhibition largely depend upon the activity of *kri-1* (Figure 3B, Supplementary Figure 2E) also consistent with DAF-16 being important.

It was particularly striking that SKN-1/Nrf2 was substantially required for lifespan extension associated with cell cycle inhibition. Mutation of *skn-1* completely blunted the beneficial effect of *cye-1/cdk-2* RNAi on longevity (Figure 3D and Supplementary Figure 2F) and therefore must be critical for the influence of cell cycle factors on aging. Genetic inactivation of cell cycle regulators led to activation of the direct SKN-1 target gene *gst-4* apparently in the intestine through *skn*-1-dependent mechanisms (Figure 4A, B and Supplementary Figure 3A-C). Moreover, RNAi knockdown of *cye-1* induced endogenous SKN-1 target gene expression (Figure 4C) supporting that the SKN-1 transcriptional response is central for cell cycle factor-mediated longevity and protection from stress. Only two recent studies indicated that SKN-1/Nrf2 is a target of germline-signaling for longevity [15, 16]. When germline stem cells are ablated (by mutation of *glp-1*), lipid-based signaling activates SKN-1 in the intestine which induces a broad transcriptional program involved in detoxification, proteasome maintenance, extracellular matrix, and lipid metabolism thereby increasing stress tolerance and longevity [15]. Diverse genetic regulatory pathways and interventions, including insulin/IGF-1, TOR, dietary restriction, mitochondrial ROS production as well as germline signaling impact on SKN-1/Nrf2 to control aging [reviewed in 17]. Our data show that cell cycle factors influence SKN-1 through germline-based signaling and further support the idea that germ cells involve SKN-1/Nrf proteins to promote longevity.

It was intriguing that we observed particularly strong effects on lifespan extension after inhibition of *cye-1* and *cdk-2.* The CYE-1/CDK-2 complex is a master regulator of the cell division cycle facilitating G1/S transition. A basic model would be that cell cycle inhibition leads to reduction of germ cell numbers and therefore the same lifespan regulating mechanisms are initiated as in germless animals. The mitotic cell cycle of *C. elegans* germ cells features a rapid progression with a highly abbreviated or absent G1 phase [26, 49]. S and G2 may thus be the major phases for regulation of cell cycle dynamics. The rapid kinetic is thought to be exerted by high CYE-1 activity during the cell cycle which may bypass G1 and drive entry into S phase. Additionally, CYE-1 is found in high levels throughout the proliferative zone [26, 50, 51]. Thus, high CYE-1 levels and an atypical cell cycle structure may be necessary to promote the pool of undifferentiated germ cells. Interestingly, other animals including flies, frogs, and zebrafish display similar characteristics in early embryonic cell divisions [reviewed in 52] suggesting evolutionarily conserved mechanisms. We assume that the potency of our tested cell cycle factors to delay aging correlates with the impact on germline proliferation. Consistent with this, disruption of *cye-1* and *cdk-2* resulted in a strongly reduced number of germ cells and sterility [27] (Supplementary Figure 1).

In addition to forward germ cell proliferation by regulating cell cycle structure per se, *cye-1/cdk-2* also contribute to the regulation of the proliferative fate versus meiotic entry decision [26, 27]. CYE-1/CDK-2 have been shown to directly phosphorylate and downregulate GLD-1 [27] which normally facilitates the switch of germ cells from mitotic into meiotic cell cycle and differentiation [53]. Moreover, GLD-1 also represses *cye-1* mRNA translation allowing a negative feedback loop [54]. Thus, *cye-1/cdk-2* may function through *gld-1* to influence the mitosis/meiosis transition. Interestingly, the ability of *cye-1/cdk-2* to influence the proliferative fate appears to be a specific function of these factors and not simply a consequence of disruption of cell cycle, as knockdown of other cell cycle factors does not induce premature meiotic entry [26]. Given that *gld-1* and likewise *glp-1* have been implicated in aging processes [7, 55], and both interact with *cye-1* and *cdk-2*, this encouraged our experimental focus on these two cell cycle factors. Possibly, these two and other cell cycle regulators might have further specific functions or yet unknown substrates in the germline directly linked to longevity and health. Notably, it was previously described that cell cycle factors, i.e. checkpoint proteins might influence lifespan by targeting postmitotic cells [29]. We show here that reduced germline proliferation is a substantial determinant for prolongation of lifespan by inhibition of cell cycle genes. Although conclusively clarifying the role of every cell cycle regulator for germline-mediated longevity will be a necessary task of future studies.

Interestingly, knockdown of not all cell cycle genes that we tested had the same impact on aging (Figure 1E). Why may this be the case? Individual cell cycle genes have differential effects in the germline [56]. In other words, cell cycle factors appear to be of dissimilar importance for maintenance of germline proliferation per se. As stated above, activity in the germline and prolongation of lifespan seem to be positively correlated. Beside *cye-1* and *cdk-2*, knockdown of the cell cycle regulators *cdk-1* and *cdc-25.1* induced the strongest lifespan extension (Figure 1E). These two genes have been shown to be critical for germline proliferation and their depletion robustly reduces the number of germ cells [26, 57-59]. *cdc-25.1* executes unique functions in the germline, while its activity is likely redundant in some somatic tissues [60]. CDK-1, in turn, is a target of CDC-25.1 and is required for mitotic and meiotic cell cycle progression as well as for oocyte maturation in the germline [59, 61, 62].

By contrast, *cdk-4*(RNAi) had very little impact on lifespan (Figure 1E), and likewise *cdk-4* only plays a subliminal role in germline proliferation in the literature [26, 59, 63]. CDK-4 acts together with CYD-1 in a canonical complex to regulate G1/S phase progression in postembryonic cells [63]. Interestingly, *cyd-1* mutants display developmental defects which are not found in *cdk-4* mutants, possibly because CYD-1 has further binding partners or CDK-4 is more stable [reviewed in 61]. Congruously with this, inhibition of *cyd-1* showed a mild but solid increase of lifespan compared to the rather weak *cdk-4*(RNAi) in our experiments. The effect was also weaker for cyclin B *cyb-1.* B-type cyclins together with Cdk1 control progression through mitosis. Three typical B-type cyclins (*cyb-1, cyb-2.1*, and *cyb-2.2*) and a distinct cyclin B3 (*cyb-3*) are expressed in *C. elegans.* It has been reported that these mitotic cyclins have partially redundant functions and only inactivation of all four cyclins fully resembles the defects in *cdk-1* inhibition [64]. Presumably, inactivation of just *cyb-1* by RNAi might therefore result in a weak *cdk-1* inhibition and longevity phenotype. Although, we cannot exclude that reduced efficiency of dsRNA-dependent inactivation of mRNA could also account for mild extension in longevity observed for these genes.

Together, our data show that inhibition of core cell cycle factors provokes stress tolerance and longevity in *C. elegans*. These effects critically involve proliferation of the germline stem cells and depend on the action of the conserved transcription factor DAF-16/FOXO, the nuclear hormone receptor DAF-12, and SKN-1/Nrf2. It will now be of interest to determine how interference with cell cycle triggers these specific germline-associated mechanisms. We propose that reduction of germ cell numbers is pivotal for cell cycle-associated longevity, but specific functions of distinct cell cycle factors in the germline as described for *cye-1/cdk-2* might also play a crucial role. By revealing that longevity benefits of germline inhibition can be conferred by modulating the cell cycle, our data also suggest that this could be instrumental for developing new methods to study germline-dependent aging so that benefits are not outweighed by detriments.

## MATERIALS AND METHODS

### *C. elegans* strains

*C. elegans* strains were grown on NGM agar plates with *E. coli* OP50 lawns [65]. Mutant strains are described in Supplementary Table S5.

### RNA interference

RNAi plasmids were picked from the Ahringer [66] or ORF-RNAi [67] libraries and confirmed by sequencing (see Supplementary Table S6). For RNAi feeding experiments cultures were grown overnight in LB-medium with 12.5μg/ml tetracycline and 50μg/ml ampicillin. The following day, cultures were diluted 1:10 in 50μg/ml ampicillin LB-medium and grown to an OD595 of 0.8-1.1. Bacteria were spun down and resuspended in 1/10 of the original volume containing 50μg/ml ampicillin and induced with 0.7mM IPTG. This culture was seeded onto NGM agar plates containing 50μg/ml ampicillin, 12.5μg/ml tetracycline and 1mM IPTG. Empty vector plasmid pPD129.36 (L4440) was used as control.

### Lifespan assays

Lifespan assays were performed unless otherwise indicated at 20°C. Animals were age-synchronized by timed egg laying and allowed to develop on control empty vector RNAi plates at 20°C. At L4 larval stage, worms were transferred on NGM RNAi plates containing 50 μM FUDR to prevent hatching of larvae. Animals were examined every day until the end of the assay. Worms that did not respond to gentle prodding with movement were scored as dead. Worms that crawled off the plates, showed extruded organs, or exploded due to internally hatching larvae were censored. All lifespans were plotted with L4 as time-point 0. Data were analyzed using a log-rank test in SigmaPlot 11.0.

To induce sterility in *glp-1(e2141)* mutants, adult animals were allowed to lay eggs at 15°C and the progeny were shifted to 25°C at L1 until L4/early adulthood. They were then placed on plates containing FUDR and RNAi bacteria for lifespan assays at 20°C. To prevent dauer formation of *daf-2(e1370), daf-12(rh61rh411)*, and *daf-9(rh50)* mutants, these strains were allowed to develop to early adulthood at 15°C before starting the lifespan at 20°C.

### Stress resistance assays

For the analysis of resistance against oxidative stress and heat, wild type worms were fed with RNAi for 4 days starting at L4 stage at 20°C. Worms were then either placed on plates containing 7.5 mM tert-butyl hydroperoxide (TBHP) or at 35°C preheated seeded RNAi plates. The criteria for analysis and censoring were essentially the same as described for lifespan assays.

### Healthspan assays

Worms were raised as described for lifespan assays. To quantify pharyngeal pumping rates, worms were examined under a dissecting microscope every other day starting at the first day of adulthood. The number of contractions of the terminal bulb was counted for 30 sec.

For thrashing assays, worms were transferred into phosphate-buffered saline and observed for thrashing movements after 5 to 10 minutes of recovery. Quantification of thrashing movements was performed on alternate days beginning on the first day of adulthood. A thrash was defined as a full change in the lateral bending direction of the whole worm corpus. Pairwise statistical comparison was performed using a Mann-Whitney U-Test.

### Brood size

Hermaphrodites were allowed to lay eggs on empty vector control. Their progeny was raised at 20°C until L4. Individual larvae were placed on fresh RNAi plates containing either empty vector control, *cye-1*(RNAi), or *cdk-2*(RNAi) and transferred on new plates every day until egg-laying ceased. The total number of progeny was counted. Pairwise statistical comparison was performed using a Mann-Whitney U-Test.

### Phenotypic analysis

Worms were fed with empty vector control, *cye-1*(RNAi), or *cdk-2*(RNAi) for two generations at 20°C. At day 2 of adulthood worms were mounted on 2% agar pads, immobilized and images were taken with a Zeiss Axioplan-2 microscope.

### Microscopy and scoring of transgenic GFP reporters

Microscopy was performed using a Zeiss Axioplan-2 microscope equipped with an AxioCam camera and AxioVision-Software Rel.4.8. Worms were placed on 2% agarose pads on slides and stunned with 2 mM levamisole. GFP was detected using an EGFP-filter set (480/20 nm excitation, 510/20 nm emission). Images were processed with AxioVision Rel 4.8 and Adobe Photoshop CS6.

Intestinal P*gst-4:*:GFP expression was assessed as described [43]. Worms were raised on RNAi until L4/young adult stage. Scoring was as follows: “high”, strong GFP levels throughout most of the intestine; “medium”, intense GFP levels anteriorly and/or posteriorly; “low”, only barely visible or no GFP expression observed.

For P*sod-3*::GFP expression L4 larvae were placed on RNAi and four days later GFP intensity was scored in the adult worms. Fluorescence intensity was categorized in “high”, worms displayed robust GFP intensity throughout their bodies or showed strong nuclear signal; “medium”, easily detectable nuclear signal at weak levels; “low”, no nuclear GFP signal observed. *p* values were determined from a Chi-square test.

### RNA isolation and quantitative PCR

For RNA preparation approximately 500 animals were grown at 20°C on empty vector or *cye-1*(RNAi) plates for 4 days starting from L4 stage. Worms were washed in M9 buffer to remove bacteria. RNA was extracted with TriReagent (Sigma), treated with DNAse (Quiagen), and purified using a RNA purification column (ZYMO Research). RNA concentration and quality was assessed using a NanoPhotometer P-Class. cDNA was synthesized with reverse transcriptase (SuperScript III First-Strand Synthesis System, Invitrogen) and oligo-dT primer. qPCR runs were per-formed in technical triplicates on a Roche Light Cycler 480 using the Takyon No Rox SYBR MasterMix blue dTTP (Eurogentec).

Samples were analyzed by the 2^−ΔΔCt^ method with normalization to the geometric mean of the reference genes *cdc-42* and Y45F10D.4. At least three biological replicates were examined for each sample. Primer sequences are listed in Supplementary Table S7.

## SUPPLEMENTARY MATERIAL FIGURES AND TABLES


