# Supplementary material for: Cell cycle controls stress response and longevity in C. elegans
Source: Aging (Albany NY). 2016 Sep 25;8(9):2100–15. doi: 10.18632/aging.101052 (PMC5076454; doi:10.18632/aging.101052)
Supplement: Supplementary file 1 [file aging-08-2100-s001.pdf]

SUPPLEMENTARY MATERIAL

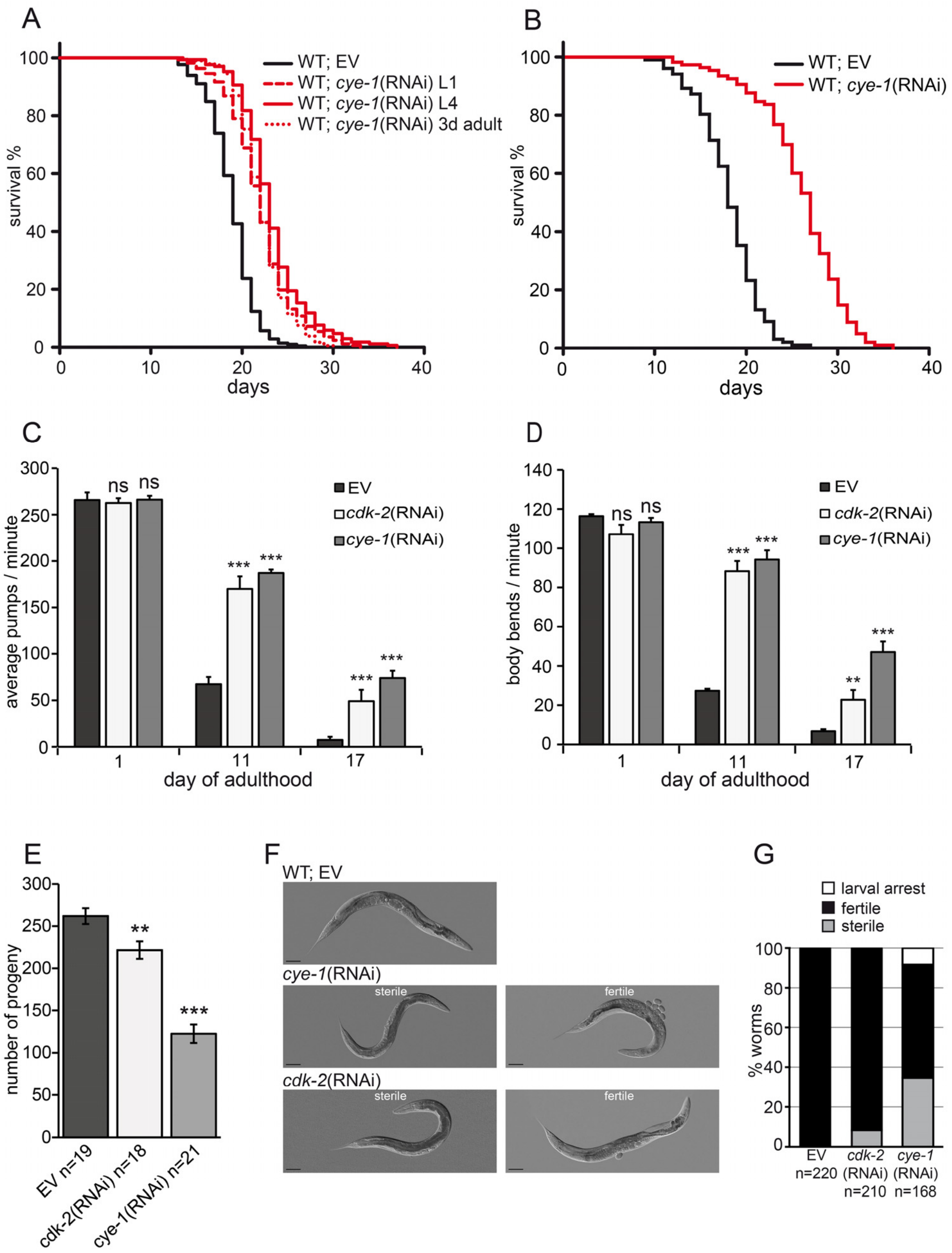

Supplementary Figure S1. (**A**) Inhibition of *cye-1* either during development or in adulthood extends lifespan. RNAi treatment was performed starting from L1 throughout life, in L4, or in adulthood (day 3). (**B**) Lifespan analysis of wild type worms treated with *cye-1*(RNAi) or empty RNAi vector without supplementation of fluorodeoxyuridine (FUDR). Knockdown of *cye-1* increases lifespan by 44%. We obtained comparable results with inclusion of FUDR. Survival plots show combined data from at least two experiments. See also Table S2 for corresponding data and statistics, and Table S1 for individual experiments. (**C**) Age-related change of pharyngeal pumping rate after knockdown of *cye-1* and *cdk-2* by RNAi. EV refers to empty RNAi vector control. The number of contractions of the terminal bulb was counted. n=10 animals per each time point. Combined data from two experiments are shown. (**D**) Knockdown of *cye-1*(RNAi) and *cdk-2*(RNAi) delayed the decline of locomotion compared to wild type on empty vector control. The number of thrashes was counted for 30 sec, n=10 animals per each time point. Pooled data from two experiments are presented. (**E**) Quantification of the progeny of wild type animals treated with empty vector control, *cye-1*(RNAi), or *cdk-2*(RNAi). RNAi feeding was performed starting from L4. Error bars indicate SEM. n, number of animals per condition. The number of progeny was significantly reduced by *cye-1*(RNAi) and *cdk-2*(RNAi) compared to wild type. ** $p < 0.01$, *** $p < 0.001$, Mann-Whitney U-Test. (**F**, **G**) *cye-1*(RNAi) and *cdk-2*(RNAi) treated animals show modest sterility or arrest development in L3 larval stage. RNAi feeding was performed for two generations. (**F**) Representative images of adult animals under the indicated conditions. Scale bar indicates 100µm. (**G**) Quantification of phenotypes.

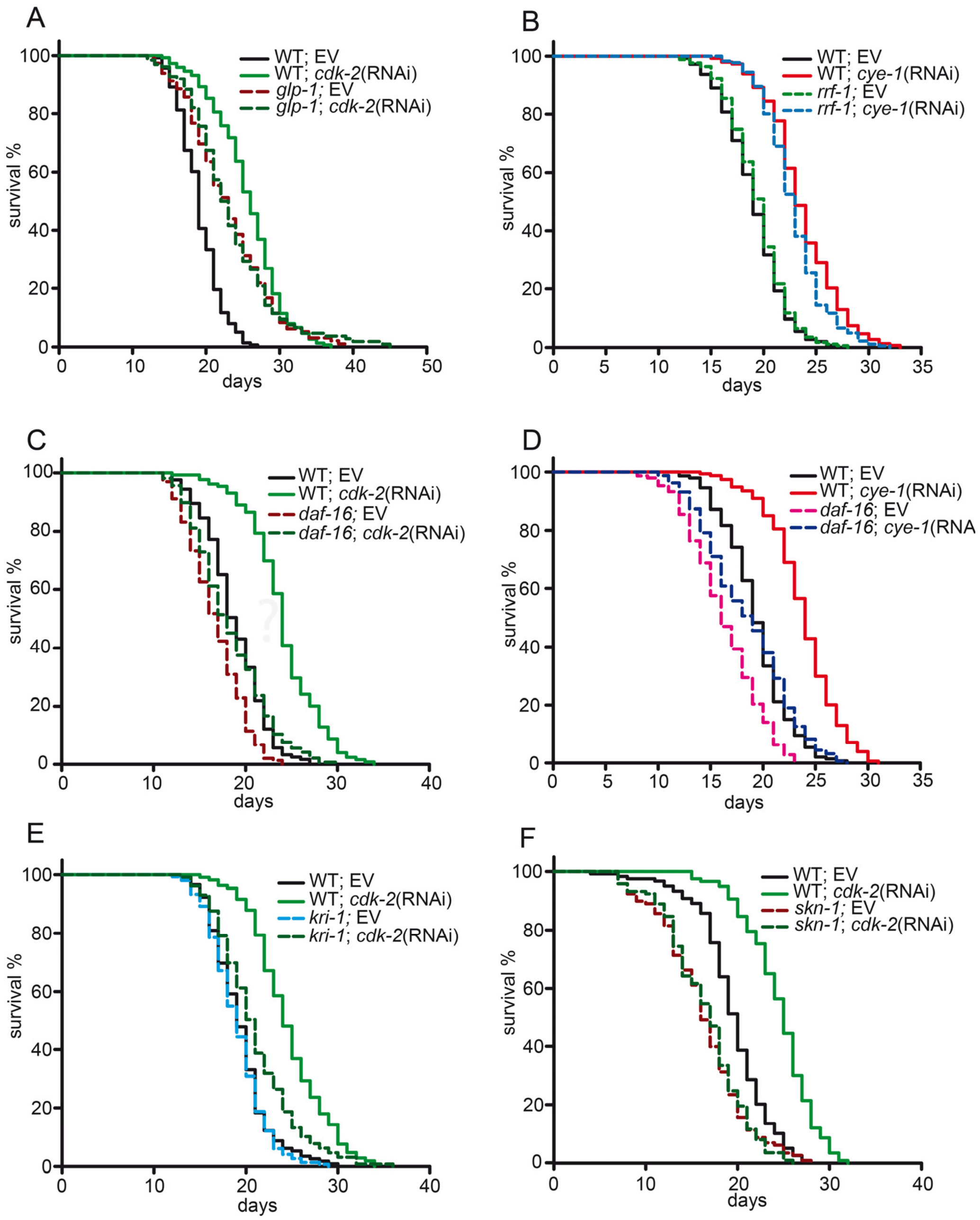

Supplementary Figure S2. (**A**) *cdk-2*(RNAi) has no effect on longevity of germless *glp-1(e2141)* mutants. *glp-1* mutants were raised at 25°C until L4/early adulthood to eliminate germ cells and then shifted to 20°C for the rest of the assay. (**B**) Effect of germline-specific RNAi knockdown of *cye-1* in *rrf-1(pk1417)* mutants. *cye-1*(RNAi) increases lifespan in *rrf-1* mutants to a similar extend as in wild type worms (18% compared to 24%, respectively). (**C**) DAF-16 is required for lifespan extension deriving from *cdk-2*(RNAi) knockdown. Survival plots showing effects of *cdk-2*(RNAi) in wild type and *daf-16(mgDf47)* mutants. (**D**) The longevity of *cye-1*(RNAi) was suppressed by *daf-16(mu86)* mutation. (**E**) KRI-1 contributes to *cdk-2*(RNAi) longevity. Survival of *cdk-2*(RNAi) worms was substantially reduced by *kri-1(ok1251)* mutation. (**F**) Importance of SKN-1 for lifespan extension deriving of *cdk-2*(RNAi). *cdk-2*(RNAi) fails to extend lifespan in the *skn-1(zu67)* mutant. These data are summarized in Supplementary Table S2 and individual experiments described in Table S1. EV refers to empty control vector.

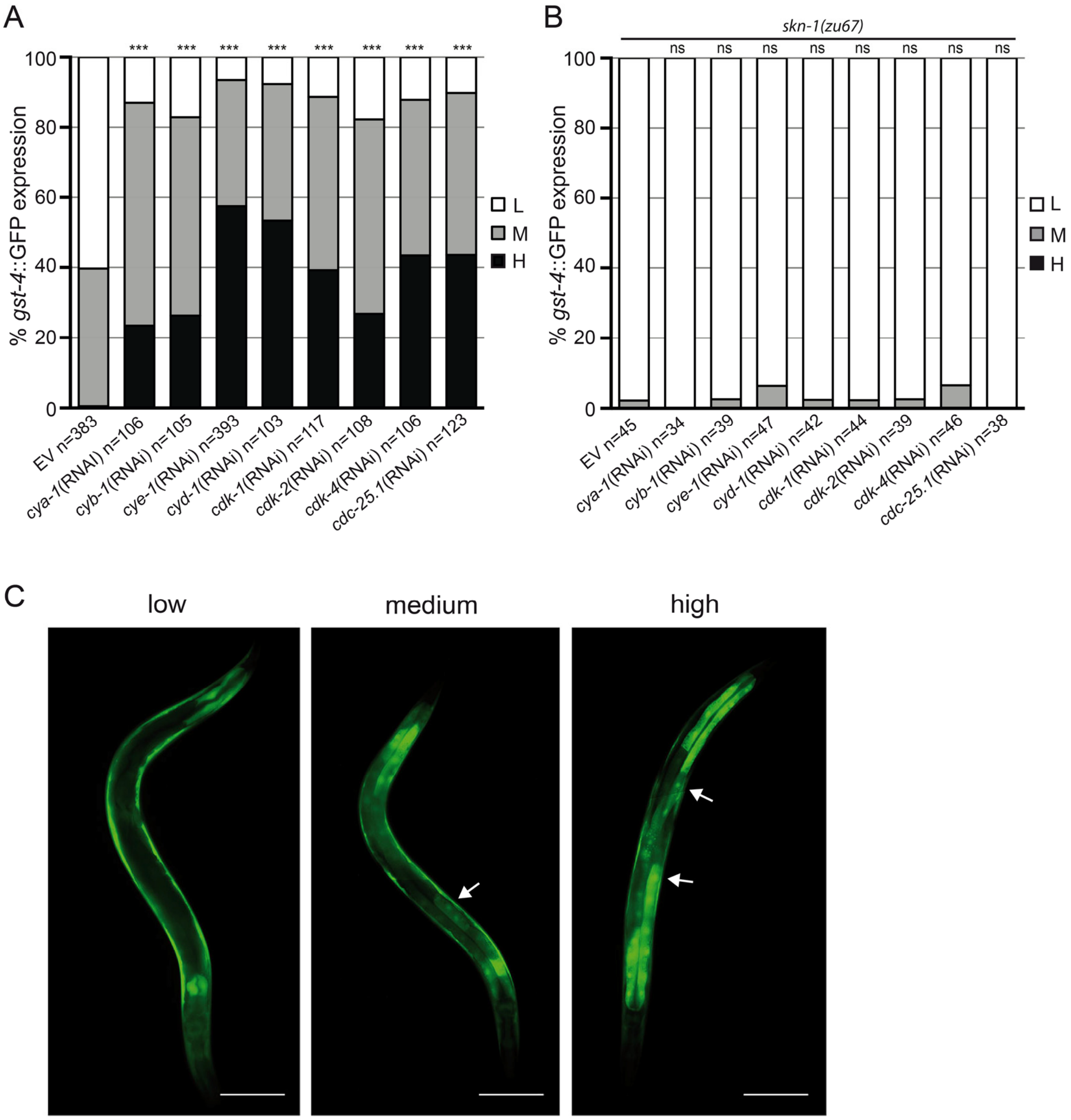

Supplementary Figure S3. (**A**) Increased expression of *Pgst-4*::GFP after RNAi knockdown of cell cycle genes. (**B**) Analysis of *Pgst-4::*GFP expression in *skn-1(zu67)* mutants after RNAi knockdown of cell cycle genes. In (**A**) and (**B**) induction of the *Pgst-4*::GFP reporter in the intestine was quantified in L4/young adult animals as low (L), medium (M), and high (H). Pooled data from at least 2 experiments. *p*-values were calculated by the Chi-square test. ****p* < 0.001. ns, not significant. n, number of worms analyzed. (**C**) Representative fluorescence images of *Pgst-4*::GFP transgenic worms quantified in low (left panel), medium (middle panel), and high (right panel). Arrows show intestinal nuclei. Scale bar represents 100 µm.

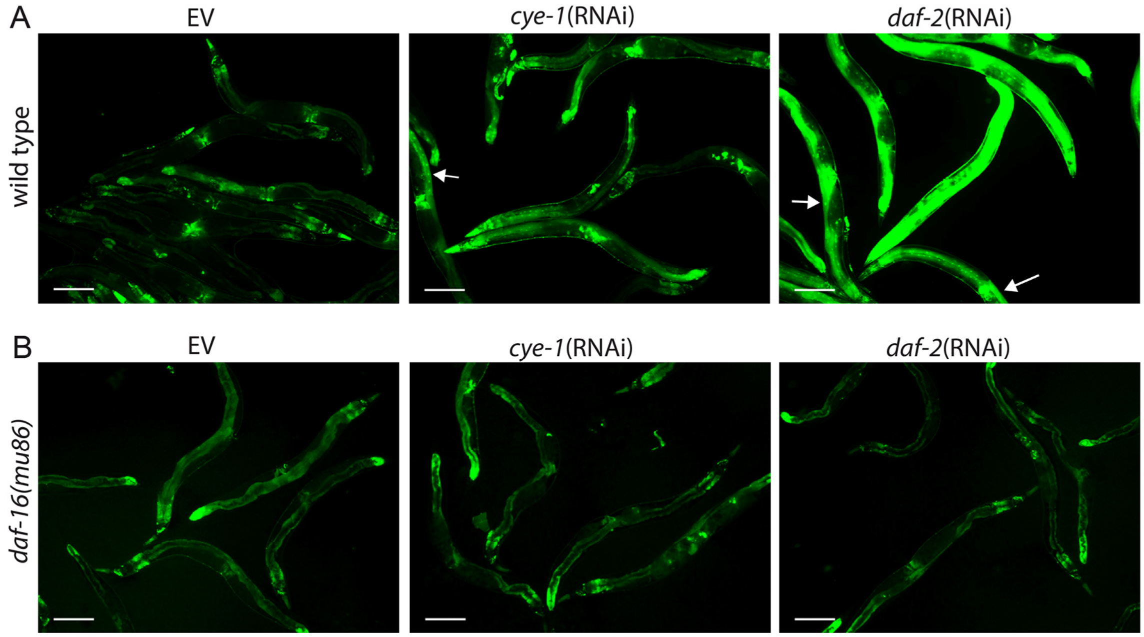

Supplementary Figure S4. (**A**) *Psod-3*::GFP expression is upregulated by *cye-1*(RNAi) knockdown. *daf-2*(RNAi) was used as positive control to examine robust *Psod-3*::GFP induction. Arrows indicate intestinal nuclear signal. (**B**) Intensity of *Psod-3*::GFP expression is greatly abolished by mutation of *daf-16*. Scale bars represent 200µm.

Supplementary Table S1. Statistical analyses of individual lifespan experiments. Data presented in Table 1 were compiled from these experiments. N represents total number of animal dying of old age versus those in total experiment. SEM, standard error of the mean. p values were calculated by log-rank.

Strain	RNAi	Mean lifespan ± SEM (days)	Median lifespan (days)	75th Percentile	25th Percentile	p value vs. N2 (control)	p value vs. mutant	% mean lifespan change vs. N2 (control)	% mean lifespan change vs. mutant	N	Figure
N2	control	18,3±0,4	18	21	16					59/65	1A + S2C
N2	*cdk-2*	25,6±0,5	26	29	23	5,3E-19		40%		59/74	1A + S2C
daf-16(mgDf47)	control	15,9±0,3	15	18	14	8,8E-05		-13%		86/103	S2C
daf-16(mgDf47)	*cdk-2*	17,3±0,5	16	20	14	0,4	6,5E-03 vs *daf-16*(ctr)	-5%	9%	89/108	S2C
N2	control	19,3±0,4	19	22	17					64/64	1A + S2C
N2	*cdk-2*	22,7±0,4	23	24	21	1,3E-10		18%		66/67	1A + S2C
daf-16(mgDf47)	control	17,9±0,3	18	20	17	1,4E-03		-7%		64/64	S2C
daf-16(mgDf47)	*cdk-2*	20±0,4	21	22	18	0,2	1,8E-05 vs *daf-16*(ctr)	4%	12%	57/59	S2C
N2	control	19,8±0,4	20	22	18					56/60	1B
N2	*cye-1*	25,5±0,7	26	30	21	1,7E-11		29%		53/60	1B
N2	control	19,2±0,4	19	21	17					58/60	1B
N2	*cye-1*	24,3±0,7	26	28	20	9,1E-09		27%		50/60	1B

N2	control	17,5±0,4	17	19	15					78/80	1E
N2	*cdk-1*	25,5±0,5	26	28	22	5,7E-24		46%		80/80	1E
N2	*cdk-4*	18,7±0,4	19	20	16	0,047		7%		75/80	1E
N2	*cya-1*	19,6±0,4	19	21	17	1,1E-03		12%		76/80	1E
N2	*cyb-1*	18,5±0,4	18	20	16	0,11		6%		78/80	1E
N2	*cyd-1*	20,6±0,5	20	23	18	3,2E-06		17%		73/80	1E
N2	*cdc-25.1*	26±0,5	26	28	23	1,4E-27		48%		78/80	1E
N2	control	18,8±0,4	18	21	16					80/81	1E
N2	*cdk-1*	26,4±0,5	26	29	25	3,7E-24		41%		77/80	1E
N2	*cdk-4*	19,7±0,4	19	22	17	0,29		5%		78/80	1E
N2	*cya-1*	20,3±0,3	20	22	18	5,7E-02		8%		79/80	1E
N2	*cyb-1*	19,8±0,3	19	22	18	0,41E		5%		79/80	1E
N2	*cyd-1*	22,3±0,5	22	25	19	4,2E-07		19%		80/80	1E
N2	*cdc-25.1*	24,2±0,4	23	27	22	7,3E-16		29%		80/80	1E
N2	control	18±0,3	18	20	16					59/60	2A
N2	*cye-1*	24,9±0,5	25	27	23	2,0E-22		38%		53/55	2A
glp-1(e2141)	control	22,9±0,3	23	24	21	3,2E-23		27%		126/145	2A
glp-1(e2141)	*cye-1*	23,8±0,4	23	26	21	1,6E-24	0,087 vs *glp-1*(ctr)	32%	4%	107/123	2A
N2	control	19,5±0,3	19	22	17					70/72	2A
N2	*cye-1*	27±0,5	28	30	25	6,6E-26		38%		68/75	2A
glp-1(e2141)	control	26,2±0,4	26	28	24	7,8E-30		34%		90/127	2A
glp-1(e2141)	*cye-1*	26,1±0,5	26	28	23	1,5E-25	0,83E vs *glp-1*(ctr)	34%	0%	88/118	2A
N2	control	18,9±0,4	19	21	17					58/60	2A + S2A
N2	*cye-1*	26,6±0,6	27	29	25	2,2E-20		41%		57/60	2A
N2	*cdk-2*	26,1±0,5	27	28	24	1,9E-19		38%		60/60	S2A
glp-1(e2141)	control	23,4±0,8	23	27	19	5,8E-08		24%		47/62	2A + S2A
glp-1(e2141)	*cye-1*	23,1±0,8	23	27	19	6,7E-07	0,81E vs *glp-1*(ctr)	22%	-1%	40/64	2A
glp-1(e2141)	*cdk-2*	23,7±0,9	23	27	20	1,6E-07	0,75 vs *glp-1*(ctr)	25%	1%	43/64	S2A
N2	control	19,3±0,3	19	21	17					80/83	2A + S2A
N2	*cye-1*	25,8±0,5	26	29	23	1,5E-22		34%		69/80	2A
N2	*cdk-2*	25,3±0,5	25	29	22	6,7E-21		31%		89/93	S2A
glp-1(e2141)	control	22,6±0,8	22	27	19	3,3E-06		17%		52/79	2A + S2A
glp-1(e2141)	*cye-1*	24,5±0,8	23	28	20	1,7E-11	0,15E vs *glp-1*(ctr)	27%	8%	70/96	2A
glp-1(e2141)	*cdk-2*	23,1±0,8	22	27	20	1,9E-08	0,8 vs *glp-1*(ctr)	20%	2%	66/88	S2A
N2	control	19,3±0,6	19	22	16					56/57	2B
N2	*cye-1*	25,2±0,6	25	28	23	1,1E-09		30%		47/54	2B
mes-1(bn7) (fertile)	control	18,4±0,5	18	21	15	0,21		-5%		54/58	2B
mes-1(bn7) (fertile)	*cye-1*	22±1,1	21	26	18	0,014	1,5E-03 vs *mes-1F*(ctr)	14%	19%	23/34	2B
mes-1(bn7) (sterile)	control	24,5±1,3	21	31	18	1,2E-04		27%		41/55	2B
mes-1(bn7) (sterile)	*cye-1*	24,3±1,3	21	31	17	1,2E-04	0,99 vs *mes-1S*(ctr)	26%	-1%	42/54	2B

N2	control	18,5±0,4	18	20	16					66/67	2B
N2	*cye-1*	23,4±0,4	24	25	21	9,1E-13		26%		58/59	2B
mes-1(bn7) (fertile)	control	18,1±0,5	17	20	15	0,94		-3%		62/64	2B
mes-1(bn7) (fertile)	*cye-1*	22,1±0,5	23	25	19	6,7E-08	4,4E-07 vs *mes-1F*(ctr)	19%	22%	61/63	2B
mes-1(bn7) (sterile)	control	23,9±0,8	24	27	21	1,4E-10		29%		47/60	2B
mes-1(bn7) (sterile)	*cye-1*	23,1±0,8	23	25	20	2,2E-08	0,66 vs *mes-1S*(ctr)	25%	-3%	48/57	2B
N2	control	20,4±0,5	20	24	18					59/60	2C+2D
N2	*cye-1*	24,3±0,5	25	26	21	7,6E-07		19%		50/66	2C+2D
daf-12 (rh61rh411)	control	18±0,4	19	20	16	1,8E-05		-12%		84/84	2C
daf-12 (rh61rh411)	*cye-1*	19,5±0,4	20	22	17	0,16	0,047 vs *daf-12*(ctr)	-4%	9%	80/87	2C
daf-9(rh50)	control	18,3±0,5	18	21	15	5,3E-03		-10%		77/79	2D
daf-9(rh50)	*cye-1*	19,5±0,6	20	23	16	0,7	1,3E-03 vs *daf-9*(ctr)	-4%	7%	70/70	2D
N2	control	19,3±0,4	19	22	17					51/51	2C+2D
N2	*cye-1*	23,6±0,5	23	25	22	5,1E-10		22%		52/52	2C+2D
daf-12 (rh61rh411)	control	17,2±0,4	17	20	15	4,6E-03		-11%		63/63	2C
daf-12 (rh61rh411)	*cye-1*	19±0,5	19	22	17	0,76	4,7E-03 vs *daf-12*(ctr)	-1%	11%	53/62	2C
daf-9(rh50)	control	18,8±0,5	19	22	16	0,99		-3%		51/51	2D
daf-9(rh50)	*cye-1*	19,9±0,6	20	23	17	0,088	0,11 vs *daf-9*(ctr)	3%	6%	53/53	2D
N2	control	20,1±0,5	20	22	17					59/60	3A+3D
N2	*cye-1*	26,7±0,6	27	29	24	6,1E-15		33%		54/60	3A+3D
daf-16(mgDf47)	control	17,5±0,5	17	20	14	6,9E-03		-13%		71/76	3A
daf-16(mgDf47)	*cye-1*	19,5±0,7	18	25	14	0,4	3,7E-03 vs *daf-16*(ctr)	-3%	12%	80/89	3A
skn-1(zu67)	control	16±0,4	16	18	14	5,2E-08		-21%		72/73	3D
skn-1(zu67)	*cye-1*	15,5±0,4	15	17	13	2,7E-10	0,44 vs *skn-1*(ctr)	-23%	-3%	63/65	3D
N2	control	18,8±0,4	19	21	16					70/74	3A+3D
N2	*cye-1*	24,2±0,4	25	27	22	1,2E-15		28%		73/76	3A+3D
daf-16(mgDf47)	control	17±0,3	17	19	14	1,9E-03		-10%		90/93	3A
daf-16(mgDf47)	*cye-1*	18,9±0,5	19	22	15	0,48	3,4E-04 vs *daf-16*(ctr)	0%	11%	88/99	3A
skn-1(zu67)	control	16,4±0,4	16	19	14	7,7E-04		-13%		60/62	3D
skn-1(zu67)	*cye-1*	16±0,5	16	19	13	5,1E-04	0,78 vs *skn-1*(ctr)	-15%	-3%	61/64	3D
N2	control	20,1±0,5	20	21	18					53/60	3B + S2E
N2	*cye-1*	27,3±0,6	28	30	25	3,3E-13		36%		41/65	3B
N2	*cdk-2*	26,3±0,6	26	30	23	6,8E-11		31%		46/66	S2E
kri-1(ok1251)	control	19,7±0,4	20	22	17	0,65		-2%		86/91	3B + S2E
kri-1(ok1251)	*cye-1*	22,5±0,6	22	26	19	3,1E-03	2,7E-05 vs *kri-1*(ctr)	12%	14%	68/72	3B
kri-1(ok1251)	*cdk-2*	22,6±0,5	22	25	20	5,3E-04	4,2E-06 vs *kri-1*(ctr)	12%	15%	73/78	S2E

N2	control	18,8±0,3	19	20	17					62/63	3B + S2E
N2	*cye-1*	24,4±0,4	24	26	22	2,2E-21		30%		72/74	3B
N2	*cdk-2*	23,3±0,4	23	25	22	3,9E-14		24%		60/60	S2E
kri-1(ok1251)	control	18,1±0,2	18	20	17	3,9E-02		-3%		63/63	3B + S2E
kri-1(ok1251)	*cye-1*	19,4±0,4	19	21	18	0,19	1,8E-03 vs *kri-1*(ctr)	4%	7%	55/59	3B
kri-1(ok1251)	*cdk-2*	19±0,4	19	20	17	0,63	0,018 vs *kri-1*(ctr)	1%	5%	56/58	S2E
N2	control	19±0,5	19	22	17					53/71	3C
N2	*cye-1*	25,7±0,7	26	29	23	2,8E-12		36%		33/63	3C
daf-2(e1370)	control	52,3±1,1	54	60	47	6,5E-42		175%		85/92	3C
daf-2(e1370)	*cye-1*	58,2±1,1	59	66	53	4,2E-46	8,0E-06 vs *daf-2*(ctr)	206%	11%	88/99	3C
N2	control	19,6±0,5	20	22	17					53/68	3C
N2	*cye-1*	24,3±0,6	24	27	22	2,3E-08		24%		42/67	3C
daf-2(e1370)	control	52,3±1,2	55	61	46	1,3E-42		167%		94/98	3C
daf-2(e1370)	*cye-1*	58,3±1,2	60	65	54	3,9E-45	3,8E-05 vs *daf-2*(ctr)	198%	11%	90/100	3C
N2	control	18,8±0,3	19	20	18					70/71	S1A
N2	*cye-1* (L1)	22±0,3	22	24	20	8,3E-14	0,94 vs L4	17%		67/68	S1A
N2	*cye-1* (L4)	21,9±0,5	22	23	20	3,0E-10		16%		46/48	S1A
N2	*cye-1* (3d adult)	20,9±0,4	21	23	19	7,2E-07	0,12 vs L4	11%		56/59	S1A
N2	control	18,4±0,2	19	20	17					76/77	S1A
N2	*cye-1* (L1)	21,6±0,3	21	23	20	1,7E-16	2,5E-03 vs L4	18%		70/74	S1A
N2	*cye-1* (L4)	22,8±0,3	23	24	21	8,1E-22		24%		62/64	S1A
N2	*cye-1* (3d adult)	21,2±0,4	21	23	19	4,9E-10	0,014 vs L4	15%		47/49	S1A
N2	control	19,6±0,4	20	21	18					65/66	S1A
N2	*cye-1* (L1)	23,1±0,4	23	25	21	2,9E-09	5,0E-03 vs L4	18%		60/64	S1A
N2	*cye-1* (L4)	24,9±0,5	24	28	22	4,9E-14		27%		62/67	S1A
N2	*cye-1* (3d adult)	23,7±0,5	23	26	21	9,1E-11	0,1 vs L4	21%		64/66	S1A
N2	control (no FUDR)	18,1±0,5	18	20	16					46/60	S1B
N2	*cye-1* (no FUDR)	25,8±0,7	25	30	23	3,9E-14		42%		49/60	S1B
N2	control (no FUDR)	18,1±0,4	18	20	16					54/59	S1B
N2	*cye-1* (no FUDR)	26,3±0,6	27	29	25	1,8E-21		45%		53/59	S1B

N2	control	19±0,4	19	21	17					65/66	S2B
N2	*cye-1*	24,2±0,4	24	26	22	3,5E-15		28%		72/73	S2B
rrf-1(pk1417)	control	19,1±0,3	19	21	17	0,89		0%		90/90	S2B
rrf-1(pk1417)	*cye-1*	23,7±0,3	24	25	22	1,3E-14	3,6E-17 vs *rrf-1*(ctr)	25%	24%	89/94	S2B
N2	control	19,1±0,3	19	21	18					80/81	S2B
N2	*cye-1*	23,1±0,4	23	26	21	4,5E-15		21%		76/77	S2B
rrf-1(pk1417)	control	19,8±0,3	20	21	18	0,2		3%		81/82	S2B
rrf-1(pk1417)	*cye-1*	22,1±0,3	22	23	20	2,0E-12	6,3E-09 vs *rrf-1*(ctr)	15%	12%	92/92	S2B
N2	control	19,5±0,4	19	21	17					74/76	S2D
N2	*cye-1*	23,7±0,3	24	25	22	4,6E-12		21%		80/80	S2D
daf-16(mu86)	control	17±0,4	17	20	15	7,5E-05		-13%		65/68	S2D
daf-16(mu86)	*cye-1*	19,5±0,4	20	22	17	0,72	7,9E-06 vs *daf-16*(ctr)	0%	14%	73/76	S2D
N2	control	19,4±0,4	19	21	18					36/37	S2D
N2	*cye-1*	24,5±0,5	25	27	23	4,2E-11		26%		40/40	S2D
daf-16(mu86)	control	16,7±0,5	16	19	14	2,7E-03		-14%		41/42	S2D
daf-16(mu86)	*cye-1*	19,1±0,7	19	23	15	0,31	1,3E-03 vs *daf-16*(crt)	-1%	14%	47/47	S2D
N2	control	18,9±0,5	19	21	16					37/39	S2D
N2	*cye-1*	23,5±0,7	23	26	21	1,2E-07		24%		34/40	S2D
daf-16(mu86)	control	14,6±0,5	15	17	12	1,9E-07		-23%		37/40	S2D
daf-16(mu86)	*cye-1*	16±0,6	15	17	13	7,4E-03	0,1 vs *daf-16*(ctr)	-15%	9%	38/40	S2D
N2	control	18,6±0,5	19	21	17					60/60	S2F
N2	*cdk-2*	23,5±0,5	24	26	21	6,7E-11		26%		57/60	S2F
skn-1(zu67)	control	14,6±0,6	15	18	12	8,7E-06		-22%		58/59	S2F
skn-1(zu67)	*cdk-2*	15,4±0,5	15	18	13	2,8E-04	0,4 vs *skn-1*(ctr)	-17%	5%	59/60	S2F
N2	control	20,4±0,5	21	23	19					59/60	S2F
N2	*cdk-2*	25,5±0,4	26	28	24	5,2E-13		25%		59/59	S2F
skn-1(zu67)	control	18±0,6	18	20	16	7,6E-03		-12%		59/60	S2F
skn-1(zu67)	*cdk-2*	17,7±0,6	18	21	16	3,6E-04	0,69 vs *skn-1*(ctr)	-13%	-2%	57/60	S2F

Supplementary Table S2. Pooled lifespan data shown in Supplementary Figure 1 and 2. SEM: standard error of the mean. 75th and 25th percentiles refer to the day at which 75% or 25% of the analyzed population is dead. N represents number of observed deaths / total number of worms in experiment. P values were obtained by log-rank.

Strain	RNAi	Mean lifespan ± SEM (days)	median lifespan (days)	75th Percentile	25th Percentile	p-value vs. N2 (control)	p-value vs. mutant	% mean lifespan change vs N2 (control)	% mean lifespan change vs mutant (control)	N	N. of Exp.	Figure
N2	control	18,9±0,2	19	20	17					211/214	3	S1A
N2	*cye-1* (L1)	22,2±0,2	22	24	21	5,5E-32	2,9E-04 vs L4	17%		197/206	3	S1A
N2	*cye-1* (L4)	23,3±0,3	23	25	21	1,6E-39		23%		170/179	3	S1A
N2	*cye-1* (3d adult)	22,1±0,3	22	24	20	5,2E-24	4,7E-03 vs L4	17%		167/174	3	S1A
N2	control (no FUDR)	18,2±0,3	18	20	16					100/119	2	S1B
N2	*cye-1* (no FUDR)	26,1±0,5	27	29	24	1,0E-33		44%		102/119	2	S1B
N2	control	19,1±0,2	19	21	17	1,0E-40				138/143	2	S2A
N2	*cdk-2*	25,6±0,4	26	29	23	2,8E-38		34%		149/153	2	S2A
glp-1(e2141)	control	23±0,6	23	27	19	9,6E-13		20%		99/141	2	S2A
glp-1(e2141)	*cdk-2*	23,4±0,6	22	27	20	2,0E-14	0,7 vs *glp-1*(ctr)	22%	1%	109/152	2	S2A
N2	control	19,1±0,2	19	21	17					145/147	2	S2B
N2	*cye-1*	23,6±0,3	23	26	22	1,4E-29		24%		148/150	2	S2B
rrf-1(pk1417)	control	19,4±0,2	20	21	17	0,37		2%		171/172	2	S2B
rrf-1(pk1417)	*cye-1*	22,9±0,2	23	25	21	5,0E-26	3,684E-24 vs *rrf-1*(ctr)	20%	18%	181/186	2	S2B
N2	control	18,8±0,3	19	21	17					123/129	2	S2C
N2	*cdk-2*	24,1±0,3	24	26	22	2,0E-27		28%		125/141	2	S2C
daf-16(mgDf47)	control	16,8±0,3	17	19	14	4,2E-07		-11%		150/167	2	S2C
daf-16(mgDf47)	*cdk-2*	18,4±0,3	18	21	15	0,99	1,3E-05 vs *daf-16*(ctr)	-2%	10%	146/167	2	S2C
N2	control	19,3±0,3	19	21	17					147/152	3	S2D
N2	*cye-1*	23,8±0,3	24	26	22	4,4E-28		23%		154/160	3	S2D
daf-16(mu86)	control	16,3±0,3	16	19	14	1,9E-11		-16%		143/150	3	S2D
daf-16(mu86)	*cye-1*	18,5±0,3	19	22	15	0,92	2,2E-08 vs *daf-16*(ctr)	-4%	14%	158/163	3	S2D
N2	control	19,4±0,3	19	21	17					115/123	2	S2E
N2	*cdk-2*	24,6±0,4	24	27	22	2,6E-21		27%		106/126	2	S2E
kri-1(ok1251)	control	19±0,2	19	21	17	0,42		-2%		149/154	2	S2E
kri-1(ok1251)	*cdk-2*	21±0,4	21	24	18	2,9E-04	2,4E-06 *kri-1*(ctr)	9%	11%	129/136	2	S2E
N2	control	19,5±0,4	20	22	18					119/120	2	S2F
N2	*cdk-2*	24,6±0,3	25	27	23	8,4E-22		26%		116/119	2	S2F
skn-1(zu67)	control	16,3±0,4	16	19	13	6,0E-06		-16%		117/119	2	S2F
skn-1(zu67)	*cdk-2*	16,6±0,4	17	19	13	1,1E-06	0,95 vs *skn-1*(ctr)	-15%	1%	116/120	2	S2F

Supplementary Table S3. Effects of *cye-1*(RNAi) and *cdk-2*(RNAi) on heat resistance (35°C). Individual stress experiments are listed, assay numbers indicate trials that were performed in parallel. Data presented in Figure 1C were compiled from these experiments. Empty RNAi vector was used for RNAi controls. T represents the total number of animals in the experiment, D represents the number of animals dead, C represents the number of animals censored at the end of the experiment. P values were obtained by log-rank.

Strain	RNAi	mean survival (hours ± SEM)	median	25th percentile	p value vs. control	T	D	C	Assay No
N2	control	10,022 ± 0,177	10	8		80	76	2	1
N2	*cye-1*	10,656 ± 0,203	12	10	2,5E-04	80	60	5	1
N2	*cdk-2*	10,848 ± 0,174	12	12	7,3E-05	80	63	4	1
N2	control	10,338 ± 0,181	10	10		80	77	0	2
N2	*cye-1*	11,137 ± 0,160	12	10	9,6E-05	80	58	5	2
N2	*cdk-2*	10,900 ± 0,128	10	10	0,048	80	72	0	2

Supplementary Table S4. Effects of *cye-1*(RNAi) and *cdk-2*(RNAi) on 7.5 mM TBHP stress tolerance. Individual stress experiments are listed, data presented in Figure 1D were pooled from these experiments. T represents the total number of animals in the experiment, D the number of animals dead, and C the number of animals censored at the end of the experiment. P values were obtained by log-rank.

Strain	RNAi	mean survival (hours±SEM)	median	25th percentile	p value vs. control	T	D	C	Assay No
N2	control	14,333 ± 0,4	14	10		80	66	4	1
N2	*cye-1*	16,605 ± 0,318	(-)	18	6,9E-09	80	31	8	1
N2	*cdk-2*	16,406 ± 0,289	18	14	1,3E-05	80	44	7	1
N2	control	12,899 ± 0,372	10	10		80	70	4	2
N2	*cye-1*	15,350 ± 0,378	18	14	8,0E-07	80	49	0	2
N2	*cdk-2*	13,832 ± 0,399	14	10	0,03	80	62	1	2

Supplementary Table S5. Strains used in this study.

Number	Internal ENH number	Genetic background	Transgene	Reference
		N2 Bristol		
CF1903	ENH381	*glp-1 (e2141) III.*		(Arantes-Oliveira and others 2002)
SS149	ENH477	*mes-1(bn7) X.*		(Strome and others 1995)
NL2098	ENH410	*rrf-1(pk1417) I.*		(Sijen and others 2001)
BR4184	ENH253	*daf-16(mgDf47) I.*		(Ogg and others 1997)
EU1	ENH 265	*skn-1(zu67)/ nT1[unc-?(n754);let-?]*		(Bowerman and others 1992)
CF2052	ENH384	*kri-1 (ok1251) I.*		(Berman and Kenyon 2006)
RG1228	ENH408	*daf-9(rh50) X.*		(Gerisch and others 2001)
AA86	ENH401	*daf-12(rh61rh411) X.*		(Gerisch and others 2001)
CB1370	ENH 77	*daf-2 (e1370) III.*		(Kenyon and others 1993)
CF1308	ENH71	*daf-16(mu86) I.*		(Lin and others 2001)
CL2166	ENH198	N2	*Is[Pgst-4::GFP*]	(Leiers and others 2003)
	ENH322	*skn-1(zu67)/nT1[unc-?]*	*Is[Pgst-4::GFP*]	
CF1553	ENH356	N2	*muIs84 [(pAD76) sod-3P::GFP + rol-6].*	(Libina and others 2003)
CF1874	ENH354	*daf-16(mu86)*	*muIs84 [(pAD76) sod-3p::GFP + rol-6].*	

Supplementary Table S6. RNAi plasmids used in this study.

Plasmid	Description
ID 6666, pPD 129.36 (L4440)	Empty RNAi vector
ID 7998, *cye-1*	ORF-RNAi library (Rual and others 2004)
ID 7997, *cdk-2*	Ahringer library (Kamath and others 2001)
ID 7286, *daf-2*	*daf-2* cDNA cloned in pPD 129.36
ID 8520, *cdk-1*	ORF-RNAi library
ID 8586, *cdk-4*	Ahringer library
ID 8521, *cya-1*	ORF-RNAi library
ID 8522, *cyb-1*	ORF-RNAi library
ID 8678, *cyd-1*	Ahringer library
ID 8517, *cdc-25.1*	ORF-RNAi library

Supplementary Table S7. Primer used for qPCR.

Gene	Name	5'->3' sequence
cdc-42	ENH 400 F<br>ENH 401 R	CTGCTGGACAGGAAGATTACG<br>CTCGGACATTCTCGAATGAAG
Y45F10.4	ENH 402 F<br>ENH 403 R	GTCGCTTCAATCAGTTCAGC<br>GTTCTTGTCAAGTGATCCGACA
gst-4	ENH 408 F<br>ENH 409 R	ATGCTCGTGCTCTTGCTGAG<br>GACTGACCGAATTGTTCTCCAT
sod-3	ENH 416 F<br>ENH 417 R	CTCCAAGCACACTCTCCCAG<br>ACCGAAGTCGCGCTTAATAGT
gcs-1	ENH_452 R<br>ENH_453 F	ATGTTTGCCTCGACAATGTT<br>AATCGATTCCTTTGGAGACC
F20D6.11	ENH_464 F<br>ENH_465 R	GGAAATTCTCGGTAGAATCGAA<br>ACGACTACGAACTTCGAACA
hmt-1	ENH479 F<br>ENH480 R	GCACTTGTCACTGTCGTT<br>GTTGTTCCAGGCAGATTCT
nit-1	ENH_466 F<br>ENH_467 R	AATCCTCCGACTATCCCTTG<br>AGCGAATCGTTTCTTTTGTG
mtl-1	ENH475 F<br>ENH476 R	GCAAGTGTGACTGCAAAA<br>AGTCTCCCTTACATCCAG
stdh-1	ENH491 F<br>ENH492 R	TGTCTTCTGTCAACCAAT<br>AATCTCCTTCTTCGTATGT
nnt-1	ENH493 F<br>ENH494 R	TGTTGGATATGTTCAAGAGG<br>TAATGGAGCGGCTGTATA
hsp-12.6	ENH497 F<br>ENH498 R	GGAGTTGTCAATGTCCTCGACG<br>GAAGTTCTCCAATGTTCTTGAC
gpd-2	ENH489 F<br>ENH490 R	GACCACATCATCTCCAAT<br>AATAATTCCGAAGTTGTCAT
